# Effect of ethanolic extracts from *Piperaceae* leaves on the reduction of skin necrosis and wound healing in an animal model of degloving injuries

**DOI:** 10.1590/acb387223

**Published:** 2023-10-30

**Authors:** Douglas Neumar Menon, Igor de Almeida Balduino Leite, Maria Theresa de Alencar Ramsdorf, Lavínia dos Santos Chagas, Sahra Amaral Arroyo, Ariany Carvalho dos Santos, Candida Aparecida Leite Kassuya, Jonas da Silva Mota, Marcio Eduardo de Barros

**Affiliations:** 1 Universidade Federal da Grande Dourados – Health Science Department – Dourados (MS) – Brazil.; 2 Universidade Federal da Grande Dourados – General Surgery – Dourados (MS) – Brazil.; 3 Universidade Estadual de Mato Grosso do Sul – Chemistry Department – Dourados (MS) – Brazil.

**Keywords:** Degloving Injuries, Ethnopharmacology, Skin, Wound Healing

## Abstract

**Purpose:**

To assess the effect of the ethanolic extract of the leaves of three species of plants from the *Piperaceae* family on reducing necrosis and enhancing wound healing in an animal model of degloving injuries.

**Methods:**

The animals were divided into six groups, each consisting of six animals: sham, negative control, EEPA (Piper *amalago* ethanolic extract), EEPG (Piper *glabratum* ethanolic extract), EEPV (Piper *vicosanum* ethanolic extract), and positive control receiving hyperbaric oxygenation. The animals underwent surgery to induce excision wounds, and the extent of cutaneous necrosis was evaluated using graphic software, while wound healing was assessed through histopathology.

**Results:**

Skin necrosis percentage area was: sham group = 62.84% 6.38; negative control group = 63.03% 4.11; P. *vicosanum* = 40.80% 4.76 p < 0.05; P. *glabratum* 32.97% 4.01 p < 0.01; P. *amalago* = 32.40% 4.61 p < 0.01; hyperbaric oxygenation = 33.21% 4.29 p < 0.01. All treated groups showed higher collagen deposition and less intense, plus predominantly mononuclear inflammatory infiltrate, suggesting improved healing process.

**Conclusions:**

The three tested extracts demonstrated efficacy in reducing the extent of cutaneous necrosis caused by degloving injuries and also showed evidence of improvement in the wound healing process.

## Introduction

Degloving injuries are most often caused by high-impact trauma, such as running over^1^. One of the mechanisms most frequently associated with degloving injuries is the gripping and rotation system, in which a body part is trapped between the vehicle wheel and the ground. In this case, friction with the moving wheel causes the skin to detach from the deep planes, leading to degloving, which can occur in the subcutaneous, subfascial, submuscular planes or even in multiple planes^2^. For these reasons, the association of degloving with trauma of multiple tissues, such as bone fractures, nerve and muscle injuries and crush syndrome are also common^3^. Thus, degloving injuries constitute a unique category of trauma because they present multiple morbidity mechanisms: the wound itself, leading to the continuity solution, tissue trauma, loss of skin coverage, devitalization of tissues, leading to increased likelihood of infection and reperfusion injury^4^.

Tissue necrosis can occur by different pathophysiological mechanisms, such as ischemia or venous congestion^5^. In the case of ischemia, cell death occurs purely and simply due to the oxygenation deficit caused by the lack of arterial blood in the affected tissue. However, necrosis occurs much more frequently due to local venous congestion^6^. As the arterial flow relies on the head of pressure, caused by ventricular systole, the arrival of arterial blood to tissues is normally easier even in partially devascularized tissues. That is the case of degloving injuries or random skin flaps. Venous return, in turn, does not rely on an active pumping by the heart, occurs passively and it is, therefore, more susceptible to total or partial flow interruptions^7^.

The fact that the wall of venous vessels is devoid of a muscular layer provides it less structural rigidity when compared to arteries, and this characteristic favors the occurrence of partial obstructions to venous drainage, due to torsion, traction or compression, narrowing the lumen of affected vessels. This condition of venous hypoflow generates accumulation of venous blood in the tissue, which generates a system of local venous hypertension, leading to vascular leakage into the interstice, further increasing the perfusion pressure that can culminate in tissue hypoperfusion at the time in which the perfusion pressure exceeds the intraluminal pressure of arteries and arterial capillaries, responsible for tissue nutrition. Similarly, any situation that leads to increased edema tends to worsen the perfusion pressure ratio, facilitating the occurrence of necrotic phenomena, a situation in which inflammation plays a relevant role. Usually, these mechanisms do not occur in isolation, especially in cases of traumatic degloving injuries, but rather there is a combination of all these conditions (ischemia, venous congestion, and inflammation/edema) reciprocally feeding each other, generating a vicious cycle in which venous hypoflow generates tissue hypoperfusion, which worsens inflammation and edema, making venous return even more difficult, culminating in the unfavorable outcome of tissue death^8^.

Reconstructive surgery often makes use of skin flaps with random vascularization, a situation in which there may be ischemic involvement of tissues. Therefore, the search for compounds or treatments that can improve the viability of these tissues becomes remarkably interesting, either by increasing blood circulation or by reducing inflammation resulting from tissue trauma^9^. It is difficult to homogenize a study group with human patients with degloving injuries due to the diversity of presentation of this type of injury. On the other hand, the low incidence of necrosis in surgically designed skin flaps makes the sample universe required for this type of study to be quite large, making clinical research in this area difficult.

In the meantime, studies with animal models have emerged as a useful tool in the search for adjuvant solutions in cases of ischemic flaps, whether surgical or traumatic. Thus, some animal models have been proposed with the aim of making this type of research possible, with emphasis on the dorsal flap suggested by McFarlane et al.^10^ and the paw degloving model described by Milcheski et al.^11^. The implementation of measures that can reduce the possibility of necrosis in flaps with questionable vascularization has several advantages in the field of reconstructive surgery, as it allows carrying out less aggressive treatments in cases of traumatic degloving injuries, reducing the possibility of tissue necrosis in random or even axial flaps, improving wound healing, and reducing the possibility of infections^12^.

Over the years, plants of the genus piper have been used as alternative treatment for toothaches, diarrhea, burns, bronchitis, infections and as wound dressing^13^. The genus Piper, of the *Piperaceae* family, is found in tropical and subtropical regions and comprises about 2,000 species. These plants are used worldwide for food and medicinal purposes^14^. Studies have shown that the genus Piper has antioxidant and anti-inflammatory effects on oxidative damage in several diseases^15^. The species of this genus are erect or climbing herbs, shrubs or rarely trees and have great economic and medicinal importance^16^. The popular use of *Piperaceae* for antibiotic, anti-inflammatory and healing purposes, as demonstrated by Durant et al.^17^, has encouraged studies on the activity of these plants in the treatment of degloving injuries, a situation in which these three characteristics can synergistically contribute to bring about beneficial results, as demonstrated by Myers^18^.

Thus, this study aimed to evaluate the effect of three plant species of the *Piperaceae* family on the reduction of tissue necrosis in an animal model of degloving injuries, based on its popular use as a healing agent in the treatment of injuries.

## Methods

This is an experimental study using adult Wistar rats from the animal facility of the Universidade Federal da Grande Dourados (UFGD), kept under a 12-hour light/dark cycle at constant temperature of 21°C with food and water ad libitum since the beginning of experiments until the day of euthanasia. The present study received approval from the ethics committee for the use of animals, under protocol number 32/2019.

### Plant materials

All species used in this study were collected in the municipality of Dourados, MS, Brazil, in the respective geographic coordinates: P. *vicosanum* Yunck. (22°12’37.8”S; 54°55’02.6”W), P. *amalago* L. (22°12’42.9”S; 54°54’55.6”W) and P. *glabratum* (Kunth) Steud. (22°12’37.7”S; 54°55’03.2”W). Identification was conducted by Elsie Franklin Guimarães, from the Research Institute of the Botanical Garden of Rio de Janeiro, RJ, Brazil. Specimens of each species were produced and deposited at the UFGD herbarium under the respective numbers: P. *vicosanum* (DDMS 4411), P. *amalago* (DDMS 4410) and P. *glabratum* (DDMS 4412). All plant names have been checked with http://www.theplantlist.org on June 8th, 2023.

### Extraction

Piper *glabratum* (1430 g), Piper *amalago* (816 g) and Piper *vicosanum* (1,100 g) leaves were dried at room temperature and subsequently submitted to three extractions by maceration in 92% ethanol, and then the extract was submitted to concentration in rotary evaporator. The extract was then dried in a hood, resulting in 203 g of Piper *glabratum* ethanolic extract (EPG) (14.19% yield), 34.27 g of Piper *amalago* ethanolic extract (EEPA) (4.2% yield) and 52.8 g of Piper *vicosanum* ethanolic extract (EEPV) (4.8% yield).

### Surgical procedure

**Figure 1:**
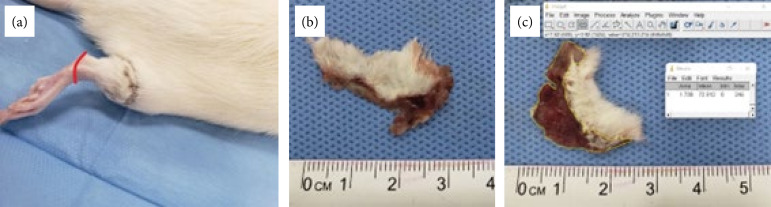
Flaps obtained after surgical procedure. **(a)** Repositioned flap showing necrosis on the 7^th^ postoperative day. The red line represents the caudal limit of the degloving injury; **(b)** Example of flap obtained after euthanasia, showing the presence of tissue necrosis. **(c)** Image exemplifying the measurement of the necrotic area using the ImageJ software, with result in cm^2^.

Source: Elaborated by the authors.

All animals underwent surgery to induce degloving injuries in the right hind limb, according to the model proposed by Milcheski et al.^11^ under general anesthesia with protocol of 60 mg/kg ketamine associated with 10 mg/kg xylazine intramuscularly applied to the left hind limb. After confirming the anesthetic plane by suppressing the corneal-palpebral reflex, the injury was induced by circumcision of the skin at the base of the limb up to the fasciocutaneous plane and subsequent traction of the flap with Allis-type gripping surgical clamps, resulting in ungloving of the limb to the level of the ankle joint. The resulting flap was then repositioned in its site of origin and sutured with 5 mononylon, thus creating a reverse and random blood flow flap (Fig. 1a).

### Drug administration

Groups were randomly divided with six animals each: negative control group, sham group, positive control group, group treated with daily sessions of hyperbaric oxygenation (HO) with 100% O_2_ and 2 ATM pressure^12^, and study groups, which respectively received: ethanolic extract from Piper *vicosanum* leaves (EEPV), ethanolic extract from Piper *glabratum* leaves (EEPG) and ethanolic extract from Piper *amalago* leaves (EEPA). Animals, with average age of 30 days and average weight of 280 g, received the respective treatments, as shown in Table 1.

**Table 1:** Summary of treatments administered according to groups.

Group (n = 6)	Treatment	Dose/route of administration
Control	0.9% Saline solution	1 mL/day – gavage
Sham	Only making the skin flap	-
Positive control	HO	30 minutes daily
Piper *vicosanum*	EEPV	300 mg/kg – gavage
Piper *amalago*	EEPA	100 mg/kg – gavage
Piper *glabratum*	EEPG	300 mg/kg – gavage

EEPA: Piper *amalago* ethanolic extract; EEPG: Piper *glabratum* ethanolic extract. EEPV: Piper *vicosanum* ethanolic extract; HO: hyperbaric oxygenation. Source: Elaborated by the authors.

Plant extracts were administered through gavage, diluted in 0.9% saline solution, totaling a volume of 1 mL. Animals from the control group received 1 mL of saline solution also by gavage, and animals from the positive control group were submitted to daily sessions of hyperbaric oxygenation with 100% oxygen at 2 ATM pressure in hyperbaric chamber specifically designed for animals^19^. The doses of respective treatments were defined based on data from literature, and doses with the best response and fewest side effects were chosen.

Animals were daily monitored with the administration of the respective treatments according to the study protocol until the seventh postoperative day, when euthanasia was performed through overdose of 100 mg/kg ketamine and 50 mg/kg xylazine, associated with exsanguination by cardiac puncture.

### Skin necrosis measurement

After euthanasia, skin flaps were removed, flattened, and photographed against a blue background (Fig. 1b). The measurement of the total area and macroscopic damaged tissue, assumed as skin necrosis area, was performed using the ImageJ software (Wayne Rasband, National Institutes of Health, United States of America, 1997) (Fig. 1c). Figure 1 shows the process of obtaining and measuring skin flaps.

### Histopathological evaluation of wound repair

The skin flaps were fixed in 10% buffered formalin. After fixation, they were cleaved, dehydrated in increasing concentrations of ethyl alcohol, cleared in xylene, and embedded in paraffin. Sections were cut at a thickness of 4 μm and stained with hematoxylin and eosin (HE) and Masson’s trichrome for assessment under light microscopy. Statistical analysis was performed using the mean ± standard error (SE), and one-criterion analysis of variance (ANOVA) followed by Tukey’s test. Results with p less than or equal to 0.05 were considered statistically significant.

## Results

### Skin necrosis reduction

**Figure 2:**
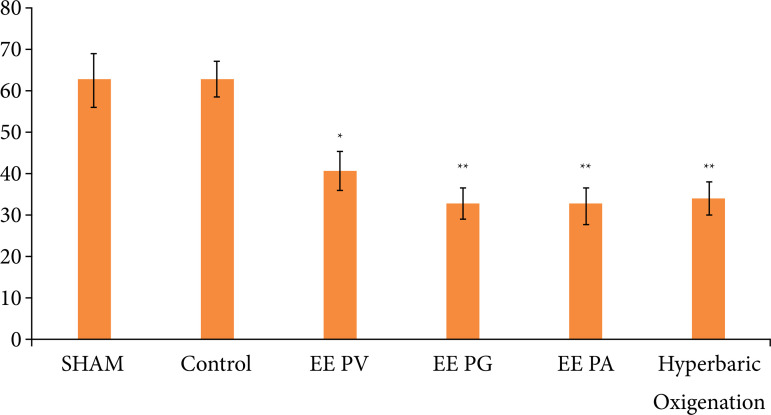
Results of means ± SE of the necrosis percentage.

SE: standard error; EEPV: Piper *vicosanum* ethanolic extract; EEPG: Piper *glabratum* ethanolic extract; EEPA: Piper *amalago* ethanolic extract; *p < 0.05; **p < 0.01. Source: Elaborated by the authors.

After euthanasia and measurement of the necrotic area of each animal, the following results were found:

- Sham group = 62.84% ± 6.38;
- Negative control group = 63.03% ± 4.11;
- P. *vicosanum* = 40.81% ± 4.76 p < 0.05;
- P. *glabratum* 32.97% ± 4.01 p < 0.01;
- *P. amalago* = 32.40% ± 4.61 p < 0.01;
- OH = 33.21% ± 4.29 p < 0.01 (Fig. 2).

**Figure 3:**
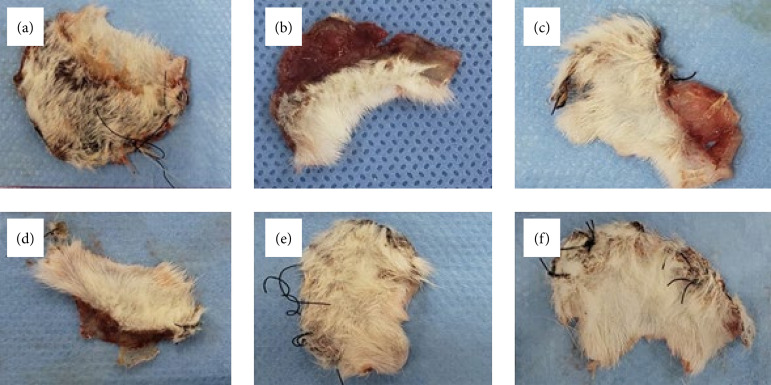
Flaps obtained after euthanasia showing different necrosis percentages: **(a)** sham group; **(b)** control group; **(c)** hyperbaric oxygenation group; **(d)** P. *vicosanum* group; **(e)** P. *glabratum* group; **(f)** P. *amalago* group.

Source: Elaborated by the authors.

Figure 3 shows examples of flaps obtained from each group. One of the animals from the sham group was excluded from the study due to the occurrence of autophagy in the necrotic flap area, impairing its measurement. The comparison of groups that used plant extracts with hyperbaric oxygenation did not show statistical difference, thus showing similarity in their results.

Table 2 shows the means ± SE of weight, total flap area, necrotic flap area and flap necrosis percentage of rats in the respective groups.

**Table 2:** Data on the weight of rats, total flap area, necrotic flap area and necrosis percentage in the respective groups.

Group (n = 6)	Animal weight (g)	Flap total area (cm^2^)	Flap necrotic área (cm^2^)	Necrosis percentage area (%)
Sham	267.83 ± 11.20	5.07 ± 0.47	3.24 ± 0.52	62.84% ± 6.38
Control	296.16 ± 12.27	3.24 ± 0.24	2.06 ± 0.24	63.03% ± 4.11
*P. vicosanum*	289.33 ± 14.03	3.47 ± 0.13	1.40 ± 0.15	40.81% ± 4.76*
*P. glabratum*	297.83 ± 14.11	3.82 ± 0.09	1.26 ± 0.16	32.97% ± 4.01**
*P. amalago*	268.66 ± 19.70	3.77 ± 0.11	1.22 ± 0.18	32.40% ± 4.61**
Hyperbaric oxygenation	264.33 ± 9.01	3.51 ± 0.14	1.17 ± 0.13	33.21% ± 4.29**

* p<0.05

** p<0.01

Source: Elaborated by the authors.

### Histopathological wound repair evaluation

**Figure 4:**
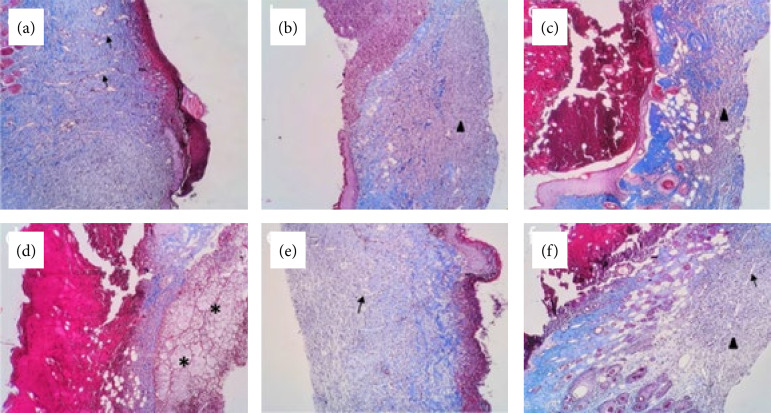
Histopathology of flaps: **(a)** control group; **(b)** sham group; **(c)** hyperbaric oxygenation group; **(d)** P. *vicosanum* group; **(e)** P. *glabratum* group; **(f)** P. *amalago* group. Masson’s trichrome, 10X caption: Angiogenesis (arrows); mononuclear inflammatory infiltrate (arrowheads); edema (asterisks).

Source: Wlaborated by the authors.

The histopathological findings are summarized in the Table 3 and Fig. 4.

**Table 3:** Histopathological findings summary.

	Reepithelization	Inflammatory infiltrate	Extracellular matrix	Angiogenesis
Sham	-	+++	+	++
Control	-	++	++	++
EEPV	-	+++	-	+
EEPG	-	++	++	++
EEPA	-	+++	+	++
HO	-	+++	+	++

EEPV: Piper *vicosanum* ethanolic extract; EEPG: Piper *glabratum* ethanolic extract; EEPA: Piper *amalago* ethanolic extract; HO: hyperbaric oxygenation. Source: Elaborated by the authors.

## Discussion

The use of natural products in conventional medicine still faces some resistance, especially in surgical areas, in which more aggressive and assertive treatments are traditionally used. However, the use of such substances for prophylactic purposes or as adjuvant in the postoperative period could lead to a better acceptance by clinicians, surgeons, and other health professionals^20^. Topical products of natural origin have already been used by surgeons to improve scar aspects, such as onion extract^21^, or to reduce edema and ecchymosis, as in the case of Arnica^22^. The development of topical products derived from Piper with a view to postsurgical use is a promising possibility, but still dependent on further studies.

The extracts used in this study were previously submitted to phytochemical analysis^23^, and the major secondary metabolites found were: alkaloids in P. *vicosanum* and P. *amalago* and organic acids, tannins, steroids, and triterpenes in P. *glabratum*. There are reports in literature of the anti-inflammatory, antioxidant, and antimicrobial activities of these secondary metabolites^24^, whose synergic action may justify the positive findings of reduced necrosis in the model used in this study. Previous studies have shown the presence of pyrrolidine amides, chalcones, flavonol^25^ and piperidines^23^ in P. *amalago*. Silva et al.^26^ demonstrated the antimicrobial activity of chalcone and flavonol has antioxidant, anti-inflammatory and antimicrobial activities^27^. Pyrrolidines are known antioxidant agents, with proven beneficial effects in improving healing in intestinal anastomoses in studies with ischemia and reperfusion models^28^^,^ 29. An extensive review conducted by Mitra et al.^30^ evaluated in details the antineoplastic action of piperidine associated with the inhibition of interleukin 6 (IL-6) production by various metabolic pathways. IL-6 is a classic pathway of inflammation, and the study of inhibitory mechanisms has been gaining prominence in the scientific community in search for anti-inflammatory therapies^31^. The anti-inflammatory activity of piperidines associated with IL-6 inhibition may be related to the effects found in this study.

In the present study, all tested plants were effective in reducing the skin necrosis percentage. The histopathological evaluation showed a slight difference in relation to the mononuclear inflammatory infiltrate present in the deep dermis, with apparent advantage for EEPG compared to EEPV and EEPA, and, although specific inflammation tests were not performed, there may be some correlation of the reduction of local inflammation with the lowest necrosis rates found in animals treated with EEPG. Other evidence of improved wound healing was observed in the histopathological analysis, particularly in the group treated with EEPG, which showed a less intense and predominantly mononuclear inflammatory infiltrate, corresponding to a more advanced phase of the inflammatory process secondary to healing. Additionally, in the treated groups, with a greater predominance in the EEPG group, there was a higher deposition of collagen fibers, supporting the hypothesis of a more advanced healing process.

The histopathological analysis did not demonstrate complete reepithelialization in any of the studied groups. However, it should be noted that this study only evaluated the first seven days, which is still a very early phase of the healing process to observe this type of effect. Observing the animals for a longer period, such as 21 to 30 days, could provide better clarification of this hypothesis. However, the main purpose of this study was to evaluate the ischemic tissue damage caused by cutaneous detachment, and extending the observation period would compromise this analysis. Future studies examining the effect of EEPG, EEPV, and EEPA on reepithelialization using this or other wound models could be highly informative.

All tested extracts showed results equivalent to those of hyperbaric oxygenation, a therapy already consolidated in the treatment of complex injuries. However, the implementation, maintenance and administration of hyperbaric therapy involves high financial costs, restricting access to this type of treatment, especially in developing countries^32^.

The necrosis produced in skin degloving injuries and in ischemic flaps is of the coagulative type due to cellular hypoperfusion. With low blood flow, the cell changes its metabolism to the anaerobic form, causing intracellular lactate accumulation and leading to changes in transmembrane transport mechanisms. The consequences are increased intracellular pH, decreased adenosine triphosphate (ATP) levels and finally coagulation of proteins, leading to cell death. Endothelial damage also leads to platelet aggregation and neutrophil adherence to the vascular wall, increasing ischemia and triggering the coagulation cascade, thus forming a vicious cycle that will culminate in cell death and tissue necrosis^33^. The anti-inflammatory activity of by-products from plants of the genus Piper can provide benefits in two different ways: reducing endothelial damage and inhibiting platelet and lymphocyte aggregation^34^.

Although endothelial damage was not evaluated in this study, it was possible to observe induction of angiogenesis and preservation of capillaries in the histopathological evaluation, suggesting the presence of this protective mechanism. Further studies analyzing endothelial damage markers may better elucidate this hypothesis.

The presence of edema in injuries is detrimental to the healing process by causing the dilution and decrease in the concentration of necessary proteins for the fibroplasia process, increasing tension at wound edges and decreasing local perfusion^35^. Previous studies^34^^,^ 36 have demonstrated the action of Piperaceae in reducing inflammation and edema, thus constituting another possible mechanism of action responsible for tissue protection.

Another important aspect is cell damage by reperfusion, which occurs when oxygen is reintroduced into the cell after a period of anaerobic metabolism. Reperfusion leads to the formation of free oxygen radicals that lead to cell death and tissue damage^37^. The antioxidant properties present in plants of the genus Piper, as described by Carsono^38^, may also exert a protective function in the case of reperfusion injury. The anti-inflammatory and antioxidant actions of Piper extracts possibly play a synergistic and multimodal role in tissue protection.

Regarding toxicity, previous studies have shown positive evidence of the low-risk potential with the use of these extracts. Acute oral toxicity, genotoxicity, and mutagenicity tests of Piper *vicosanum* have demonstrated the safety of this plant^36^. On the other hand, a study conducted by Stein et al.^39^ demonstrated signs of subacute toxicity in tests with Piper *amalago*, showing reduction in hematocrit, increase in platelet counts and serum levels of cholesterol and alkaline phosphatase, although showing safety from the point of view of genotoxicity or mutagenicity during comet and micronucleus tests. The authors suggested the possibility of toxicity with the extended use of this substance. Therefore, the use for short periods of time, such as for the acute treatment of injuries, can still be considered after the conduction of these studies. Finally, tests with Piper *glabratum* did not show signs of acute or subacute oral toxicity^34^.

The results obtained in this study in future clinical trials could offer efficient therapeutic alternatives at substantially reduced costs, expanding access to treatment, especially in underdeveloped or developing regions, while the offer of expensive treatments, such as hyperbaric oxygenation and vacuum therapy, is still low in many countries.

## Conclusion

All plants under study showed beneficial potential in reducing necrosis in the present model. A comparison of plant extracts with positive control, hyperbaric oxygenation^12^, known to be effective in reducing tissue necrosis in situations of circulatory compromise, showed statistically equivalent results, but from the economic point of view the adoption of therapies derived from *Piperaceae* may be more advantageous. The low financial cost associated with these products is a promising alternative for prophylactic or adjuvant therapies in surgical procedures or wound treatment, especially in low-income countries.

The literature demonstrates safety from the point of view of mutagenicity and genotoxicity for all extracts under study, but there may be subacute oral toxicity with the use of Piper *amalago*, and its use for extended periods must be carefully analyzed.

Thus, *Piperaceae* seems to be promising alternatives for the future development of therapies for compromised circulatory conditions, as well as for the treatment of complex wounds, and further studies are still needed.

## Data Availability

All data sets were generated or analyzed in the current study.

## References

[1] Milcheski DA, Ferreira MC, Nakamoto HA, TUMA P, Gemperli R (2010). Tratamento cirúrgico de ferimentos descolantes nos membros inferiores: proposta de protocolo de atendimento. Rev Col Bras Cir.

[2] Xu Q, Zhu L, Wang G, Sun Y, Wang J, Lin J, Pei Y, Cui Y, Liu B, Yuan X, Zhang H, Zang C. (2022). Application of cryopreserved autologous skin replantation in the treatment of degloving injury of limbs. J Plast Reconstr Aesthet Surg.

[3] Boudreault DJ, Lance SH, Garcia JA (2016). Barbed Suture as a Treatment Approach in Complex Degloving Injuries. Ann Plast Surg.

[4] Sakai G, Suzuki T, Hishikawa T, Shirai Y, Kurozumi T, Shindo M. (2017). Primary reattachment of avulsed skin flaps with negative pressure wound therapy in degloving injuries of the lower extremity. Injury.

[5] Covello GS, Martins DVR, Padilha GC, Cavalheiro CS, Vieira LA, Caetano EB (2022). Serratus anterior muscle flap for reconstruction of extremity injuries. Acta Ortop Bras.

[6] Mousavian A, Sabzevari S, Parsazad S, Moosavian H. (2022). Leech Therapy Protects Free Flaps against Venous Congestion, Thrombus Formation, and Ischemia/Reperfusion Injury: Benefits, Complications, and Contradictions. J Bone Joint Surg Am.

[7] Qiao Q, Moon W, Zhang F, Chen SG, Kunda L, Lineaweaver WC, Buncke HJ (1999). Patterns of flap loss related to arterial and venous insufficiency in the rat pedicled TRAM flap. Ann Plast Surg.

[8] Maitani K, Tomita K, Tashima H, Nomori M, Taminato M, Kubo T. (2020). Successful Salvage of Pedicled Latissimus Dorsi Flap after Venous Thrombosis by Selective Thrombolytic Therapy. Plast Reconstr Surg Glob Open.

[9] Habibi Z, Hoormand M, Banimohammad M, Ajami M, Amin G, Pazoki-Toroudi H. (2022). The Novel Role of Crocus sativus L. in Enhancing Skin Flap Survival by Affecting Apoptosis Independent of mTOR: A Data-Virtualized Study. Aesthetic Plast Surg.

[10] McFarlane RM, DeYoung G, Henry RA (1965). The design of a pedicle flap in the rat to study necrosis and its prevention. Plast Reconstr Surg.

[11] Milcheski DA, Nakamoto HA, Tuma P, Nóbrega L, Ferreira MC (2013). Experimental Model of Degloving Injury in Rats. Ann Plast Surg.

[12] Menon DN, Teixeira L, Paurosi NB, Barros ME. (2017). Effects of heparin and hyperbaric oxygenation on necrosis reduction in an animal model for degloving injuries. Rev Col Bras Cir.

[13] Zakaria ZA, Patahuddin H, Mohamad AS, Israf DA, Sulaiman MR (2010). In vivo anti-nociceptive and anti-inflammatory activities of the aqueous extract of the leaves of Piper sarmentosum. J Ethnopharmacol.

[14] Pamar V, Jain S, Bisht K, Jain R, Taneja P, Jha A, Tyagi O, Prasad A, Wengel J, Olsen C, Boll P. (1997). Phytochemistry of the genus Piper. Phytochemistry.

[15] Soares KD, Bordignon SA, Apel MA (2022). Chemical composition and anti-inflammatory activity of the essential oils of Piper gaudichaudianum and Piper mikanianum. J Ethnopharmacol.

[16] Negreiros JRS, Miqueloni DP (2015). Morphological and phytochemical characterization of Piper hispidinervum DC. and Piper aduncum L. populations in the state of Acre. Rev Ceres.

[17] Durant-Archibold AA, Santana AI, Gupta M (2018). Ethnomedical uses and pharmacological activities of most prevalent species of genus Piper in Panama: A review. J Ethnopharmacol.

[18] Myers MB. (1986). Understanding Flap Necrosis. Plast Reconstr Surg.

[19] Bertoletto PR, Fagundes DJ, Simões MDJ, Oshima CT, Montero EFDS, Simões RS, Fagundes ATN (2007). Effects of hyperbaric oxygen therapy on the rat intestinal mucosa apoptosis caused by ischemia-reperfusion injury. Microsurgery.

[20] Patel N, Pierson J, Lee T, Mast B, Lee BTB, Estores I, Singhal D. (2017). Utilization and Perception of Integrative Medicine Among Plastic Surgery Patients. Ann Plast Surg.

[21] Tran B, Wu JJ, Ratner D, Han G. (2020). Topical Scar Treatment Products for Wounds: A Systematic Review. Dermatol Surg.

[22] Ruan QZ, Chen AD, Tran B, Epstein S, Fukudome EY, Tobias AM, Lin SJ, Lee BT, Yeh GY, Singhal D. (2019). Integrative Medicine in Plastic Surgery. Ann Plast Surg.

[23] Oesterreich SA, Traesel GK, Piccinelli AC, Aquino DFS, Mota J, Estanislau C, Kassuya CAL (2015). Antidepressant and anxiolytic effects of ethanol extracts from four piper species. SaBios Rev Saúde Biol.

[24] Salehi B, Zakaria ZA, Gyawali R, Ibrahim SA, Rajkovic J, Shinwari ZK, Khan T, Sharifi-Rad J, Ozleyen A, Turkdonmez E, Valussi M, Tumer TB, Fidalgo LM, Martorell M, Setzer WN (2019). Piper Species: A Comprehensive Review on Their Phytochemistry, Biological Activities and Applications. Molecules.

[25] Novaes AJ, Barison A, Veber CL, Negrão FJ, Kassuya CAL, de Barros ME (2014). Diuretic and antilithiasic activities of ethanolic extract from Piper amalago (Piperaceae). Phytomedicine.

[26] Silva J, Rocha JE, Cunha Xavier, Freitas TS, Coutinho HDM, Bandeira PN, Oliveira MR, Rocha MN, Marinho Kassio, Ribeiro LR, Menezes RRPPB, Marinho MM, Teixeira AMR, Santos HS, Marinho ES (2022). Antibacterial and antibiotic modifying activity of chalcone (2E)-1-(4j-aminophenyl)-3-(4-methoxyphenyl)-prop-2-en-1-one in strains of Staphylococcus aureus carrying NorA and MepA efflux pumps: In vitro and in silico approaches. Microb Pathog.

[27] Cazarolli L, Zanatta L, Alberton E, Figueiredo MSB, Folador P, Damazio R, Pizzolatti M, Silva FRB (2008). Flavonoids: Prospective Drug Candidates. Mini Rev Med Chem.

[28] Teke Z, Aytekin FO, Kabay B, Yenisey C, Aydin C, Tekin K, Sacar M, Ozden A. (2007). Pyrrolidine Dithiocarbamate Prevents Deleterious Effects of Remote Ischemia/Reperfusion Injury on Healing of Colonic Anastomoses in Rats. World J Surg.

[29] Teke Z, Kabay B, Aytekin FO, Yenisey C, Demirkan NC, Sacar M, Erdem E, Ozden A. (2007). Pyrrolidine dithiocarbamate prevents 60 minutes of warm mesenteric ischemia/reperfusion injury in rats. Am J Surg.

[30] Mitra S, Anand U, Jha NK, Shekhawat MS, Saha SC, Nongdam P, Rengasamy KRR, Proćków J, Dey A. (2021). Anticancer Applications and Pharmacological Properties of Piperidine and Piperine: A Comprehensive Review on Molecular Mechanisms and Therapeutic Perspectives. Front Pharmacol.

[31] Yeung YT, Aziz F, Guerrero-Castilla A, Arguelles S. (2018). Signaling Pathways in Inflammation and Anti-inflammatory Therapies. Curr Pharm Des.

[32] Thiruvoth FM, Rajasulochana S, Kumar SM, Saravanan E, Sivanantham P, Kar SS (2022). Hyperbaric oxygen therapy as an adjunct to the standard wound care for the treatment of diabetic foot ulcers in Indian patients: a cost utility analysis. Expert Rev Pharmacoecon.

[33] D’Arcy MS (2019). Cell death: a review of the major forms of apoptosis, necrosis and autophagy. Int J Cell Biology.

[34] Branquinho LS, Santos JA, Cardoso Silva, Mota J, Junior UL, Kassuya CAL, Arena AC (2017). Anti-inflammatory and toxicological evaluation of essential oil from Piper glabratum leaves. J Ethnopharmacol.

[35] Myers MB. (1967). The Effect of Edema and External Pressure on Wound Healing. Arch Surg.

[36] Brait DRH, Vaz MS, Silva Arigo, Carvalho LNB, Araújo FHS, Vani JM, Silva Mota, Cardoso CAL, Oliveira RJ, Negrão FJ, Kassuya CAL, Arena AC (2015). Toxicological analysis and anti- inflammatory effects of essential oil from Piper vicosanum leaves. Regul Toxicol Pharmacol.

[37] Hauet T, Pisani DF (2022). New Strategies Protecting from Ischemia/Reperfusion. Int J Mol Sci.

[38] Carsono N, Tumilaar SG, Kurnia D, Latipudin D, Satari MH (2022). A Review of Bioactive Compounds and Antioxidant Activity Properties of Piper Species. Molecules.

[39] Stein J, Jorge BC, Reis ACC, Radai JAS, da Silva Moreira S, Fraga TL, da Silva Mota J, Oliveira RJ, Kassuya CAL, Arena AC (2022). Evaluation of the safety of ethanolic extract from Piper amalago L. (Piperaceae) leaves in vivo: Subacute toxicity and genotoxicity studies. Regul Toxicol Pharmacol.

